# The Book World of Medicine and Science

**Published:** 1893-04-22

**Authors:** 


### The Book World of Medicine and Science.

### BOOK REVIEWS.

Hygiene and Public Health By B. Arthur Whitelegge, M.B., B.Sc.Lond., D.P.H.Camb. (London: Cassell and Company, Limited.)

Among the many books on this subject which have appeared in recent years, this manual by Dr. Whitelegge has always taken a very high place, and we are glad to be able to welcome a second edition. Several additions and alterations have been found necessary in order to bring it up to date, and more especially is this the case in the chapter on Sanitary Law, as within the last two years several important public health measures have been passed. In the chapter on air, want of space has deterred Dr. Whitelegge from entering into details regarding many of the diseases produced by aërial impurities. He states that colliers do not, comparatively speaking, suffer much from phthisis, and that their death-rate from this disease is comparatively low. If by phthisis he means tubercular disease, we are inclined to agree with him, but, on the other hand, if he includes in this other diseases of the lungs, such as bronchitis, emphysema, and bronchiectasis, we have, after a large experience and after a careful examination of several thousand deaths of coal miners, come to the conclusion that this statement is based on insufficient data and that the death-rate from respiratory diseases in colliers is comparatively high. In dealing with artificial ventilation, Dr. Whitelegge does not mention the method brought out by Mr. Key, which has been found to give very satisfactory results. The chemical examination of water is very concisely treated, but one of the simplest processes for the estimation of the nitrites and nitrates is not given—viz., Crum's method by means of the nitrometer. In regard to the important question of lead in water and its cause, we agree with Dr. Whitelegge that "more evidence is needed before any final conclusion can be arrived at, and it is at least probable that the mischief may not always be due to the same cause." There can be little doubt that the presence of an acid, either organic or inorganic, is in most instances an important factor in its causation. The subject of food is dealt with very completely, although students could not depend on the analytical part for examination purposes. In regard to the question of milk in relation to disease we gather from the text that the author is a believer in bovine scarlet fever, but he also candidly states that the theory is rejected by the veterinary profession, and by some medical authorities. We do not think it has yet been proved that scarlet fever, or a fever capable of producing it in the human being, does exist in the cow. Very capable observers have all along held that in these outbreaks, all sources of human contamination had not been rigidly excluded. Chapter VI., on buildings generally and on hospitals and schools is very well arranged, and in the space occupied much valuable information is given. In speaking of construction of hospitals for infectious diseases, we are glad to see that Dr. Whitelegge says that the "proper function of temporary hospitals is to supplement, and not to supersede, permanent buildings of brick or stone." Another remark which local authorities would do well to think over is that the economy in first cost is usually exaggerated, and that an annual increasing outlay is required to keep them in repair. Their only advantage is, as Dr. White-

THE BOOK WORLD OF MEDICINE AND SCIENCE.—(*continued*).

legge says, that they can be rapidly erected. A little more space might have been devoted to the very important subject of school hygiene, and we trust that this will be remedied in another edition. For the chapter on removal of refuse, &c., we have nothing but the highest praise to give, and we would specially call attention to the statements contained at pages 188 to 192 on the comparative advantages of water carnage and dry systems. Sewage disposal is treated of more meagrely than one might have anticipated. In no other text-book on hygiene is the subject of disinfection dealt with in a more sanitary manner; the advantages of disinfection by heat are stated in a clear way, and the truth about chemical disinfection is tersely given. Chapter XV. gives a summary of sanitary law, and includes the Public Health Act (1875), Factory and Workshop Acts, Housing of the Working Classes Act (1890), Infectious Disease Prevention Act (1890), and the Public Health (London) Act (1891). The main provisions of these Acts are given. Vital statistics, a subject with which the author is thoroughly familiar, are then dealt with Dr. Whitelegge has succeeded in putting this subject in a very clear manner. We can thoroughly recommend Dr. Whitelegge's work to students and medical practitioners.

